# Author Correction: NS2 induces an influenza A RNA polymerase hexamer and acts as a transcription to replication switch

**DOI:** 10.1038/s44319-024-00279-3

**Published:** 2024-10-07

**Authors:** Junqing Sun, Lu Kuai, Lei Zhang, Yufeng Xie, Yanfang Zhang, Yan Li, Qi Peng, Yuekun Shao, Qiuxian Yang, Wen-Xia Tian, Junhao Zhu, Jianxun Qi, Yi Shi, Tao Deng, George F Gao

**Affiliations:** 1 College of Veterinary Medicine, Shanxi Agricultural University, Jinzhong, 030801 China; 2 Shanxi Academy of Advanced Research and Innovation, Taiyuan, 030032 China; 3 CAS Key Laboratory of Pathogen Microbiology and Immunology, Institute of Microbiology, Chinese Academy of Sciences, Beijing, 100101 China; 4 Institute of Pediatrics, Shenzhen Children’s Hospital, Shenzhen, 518026 China; 5 Department of Basic Medical Sciences, School of Medicine, Tsinghua University, Beijing, 100084 China; 6 International Institute of Vaccine Research and Innovation (iVac), Savaid Medical School, University of Chinese Academy of Sciences, Beijing, 100049 China

## Abstract

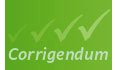

**Correction to:** *EMBO Reports* (2024). 10.1038/s44319-024-00208-4 | Published online 18 July 2024

**The authorship of the manuscript is corrected**.

**An Author affiliation is corrected**.

**Omitted grants are corrected in the author acknowledgments section**.

Wen-Xia Tian was omitted as a co-corresponding author. This is corrected.

The affiliations for George F Gao are corrected. The following affiliation is added. ^1^College of Veterinary Medicine, Shanxi Agricultural University, Jinzhong 030801, China.

The author acknowledgements section is corrected from:

Acknowledgements

We thank all staff at the cryo-EM Center, Shanxi Academy of Advanced Research and Innovation for their technical supports on the cryo-EM data collection. The study was supported by the National Key Research and Development Program of China (2022YFF1203200 to TD and 2021YFC2300700 to YS and TD), Strategic Priority Research Program of CAS (XDB29010000 to GFG and YS), National Natural Science Foundation of China (NSFC) (81871658, 32192452, 32100119, 31870160, and 32070173 to YS, QP, YL, and TD), and Beijing Natural Science Foundation (M22031 to TD).

To: (Changes in bold)

Acknowledgements

We thank all staff at the cryo-EM Center, Shanxi Academy of Advanced Research and Innovation for their technical supports on the cryo-EM data collection. The study was supported by the National Key Research and Development Program of China (2022YFF1203200 to TD and 2021YFC2300700 to YS and TD), Strategic Priority Research Program of CAS (XDB29010000 to GFG and YS), National Natural Science Foundation of China (NSFC) (81871658, 32192452, 32100119, 31870160, and 32070173 to YS, QP, YL, and TD), **Shanxi Key R&D Program (202102130501001 to WXT), The Earmarked Fund for Shanxi Agriculture Research System (2023-07, 2024CYJSTX15 to WXT) and The Special Fund for Science and Technology Innovation Teams of Shanxi Province (202204051001022 to WXT)** and Beijing Natural Science Foundation (M22031 to TD).

All authors agree to this author correction.

The original article has been corrected.

