# Supplementary material for: NS2 induces an influenza A RNA polymerase hexamer and acts as a transcription to replication switch
Source: EMBO Rep. 2024 Jul 18;25(11):8. doi: 10.1038/s44319-024-00208-4 (PMC11549089; doi:10.1038/s44319-024-00208-4)
Supplement: Supplementary file 1 — Appendix [file 44319_2024_208_MOESM1_ESM.pdf]

Appendix for: NS2 induces an influenza A RNA polymerase hexamer and acts as a transcription to replication switch

Appendix Table of Contents

Page 3 Appendix Figure S1

Page 4 Appendix Figure S2

Page 5 Appendix Figure S3

Page 6 Appendix Figure S4

Page 7 Appendix Figure S5

Page 8 Appendix Table S1

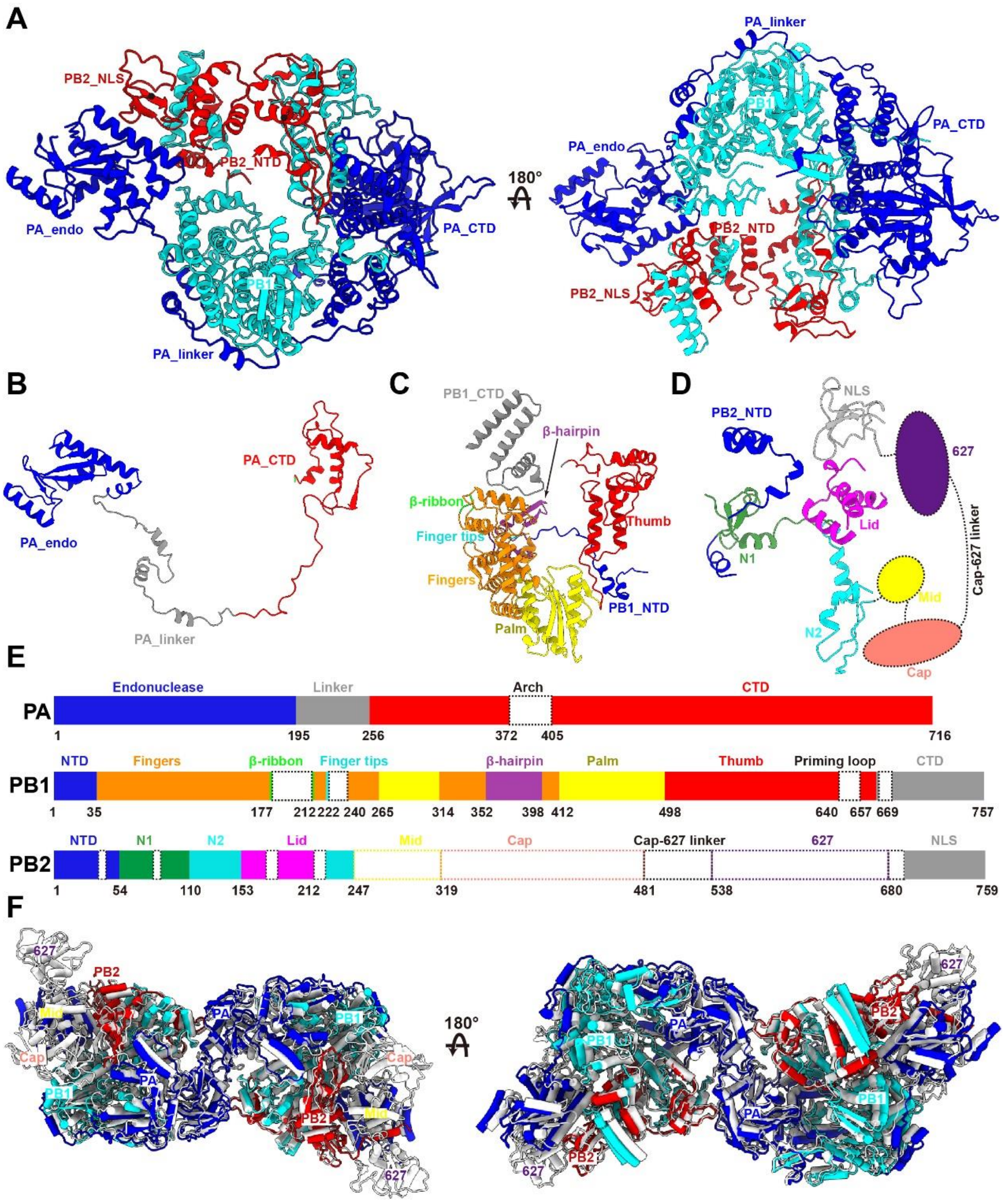

Appendix Figure S1. Structure of FluAPol heterotrimer.

(A) The structure of a polymerase protomer in FluAPol-NS2 complex. (B-D) The structures of polymerase subunits. (E) Schematic diagram of domains structure of each of polymerase subunits. The unmodelled regions were indicated as dashed squares. (F) Overlay of structures of the dimeric polymerase heterotrimer from FluAPol-NS2 complex and apo H3N2 polymerase. The two structures could be well superimposed, except the Mid, Cap, Cap-627 linker and 627 domains which are disordered in FluAPol-NS2 complex.

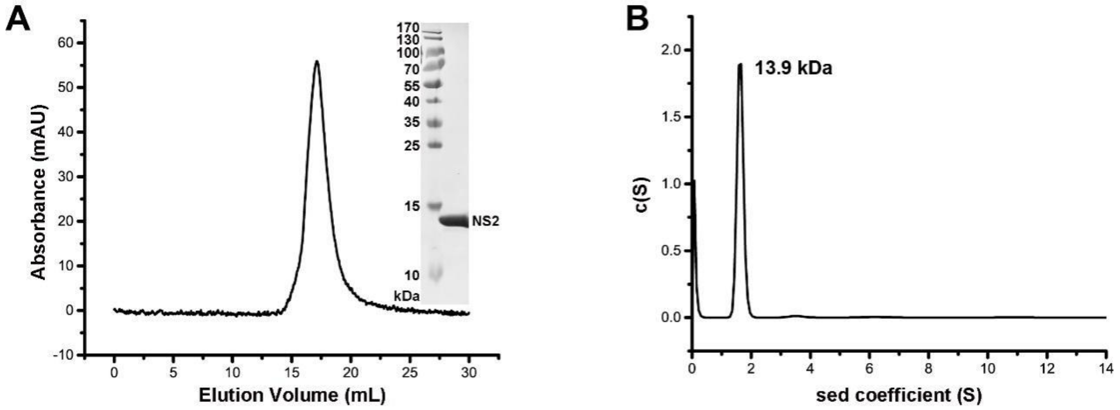

Appendix Figure S2. Aggregated form of full-length NS2 in solution.

(A) Size-exclusion chromatography and SDS-PAGE gel profiles of full-length NS2. (B) Analytical ultracentrifugation analysis of full-length NS2. These results showed that the NS2 exists as monomer in solution.

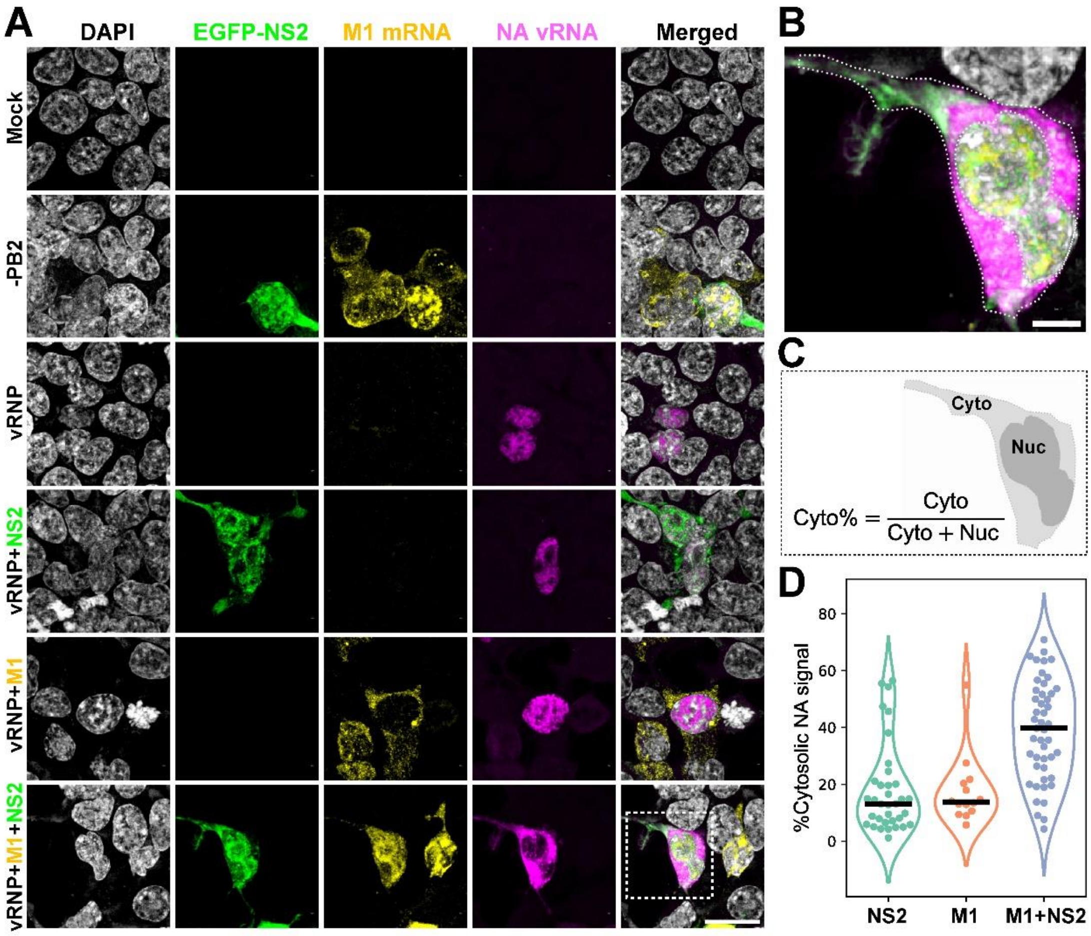

Appendix Figure S3. The vRNPs are restricted in the nucleus when NS2 is expressed alone.

(A) HEK-293T cells were transfected with plasmids as indicated for 36 h and then probed against NA vRNA and M1 mRNA using Cy5 and Cy3 labeled single-molecule inexpensive FISH (smiFISH) probes. DAPI marks the cellular nuclei. Mock, no transfection. -PB2, the expression plasmids of M1, NS2 and vRNP except for PB2 protein were co-transfected. vRNP, the expression plasmids of vRNP were co-transfected. vRNP+NS2, the expression plasmids of vRNP and NS2 proteins were co-transfected. vRNP+M1, the expression plasmids of vRNP and M1 proteins were co-transfected. vRNP+M1+NS2, the expression plasmids of vRNP, M1, and NS2 proteins were co-transfected. Scale bar, 20 µm. (B) Zoomed in view of the vRNP+NS2+M1 co-transfection group, as marked by the dashed rectangle in (A). Scale bar, 5 µm. (C) The efficiency of nuclear export of vRNP is determined by the ratio of vRNA in the cytosolic (Cyto%). (D) Quantification of the cytosolic fraction of vRNAs (Cyto%) of cells from the vRNP+NS2, the vRNP+M1, or the vRNP+M1+NS2 co-transfection groups.

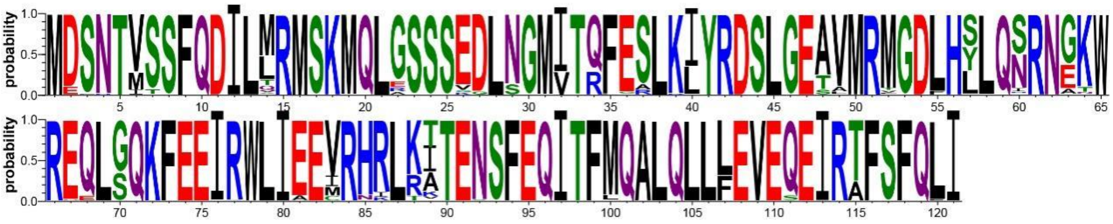

Appendix Figure S4. Conservatism of influenza A NS2.

The sequence alignment was performed using all the available sequences of influenza A NS2, which suggested that the critical residues were highly conserved.

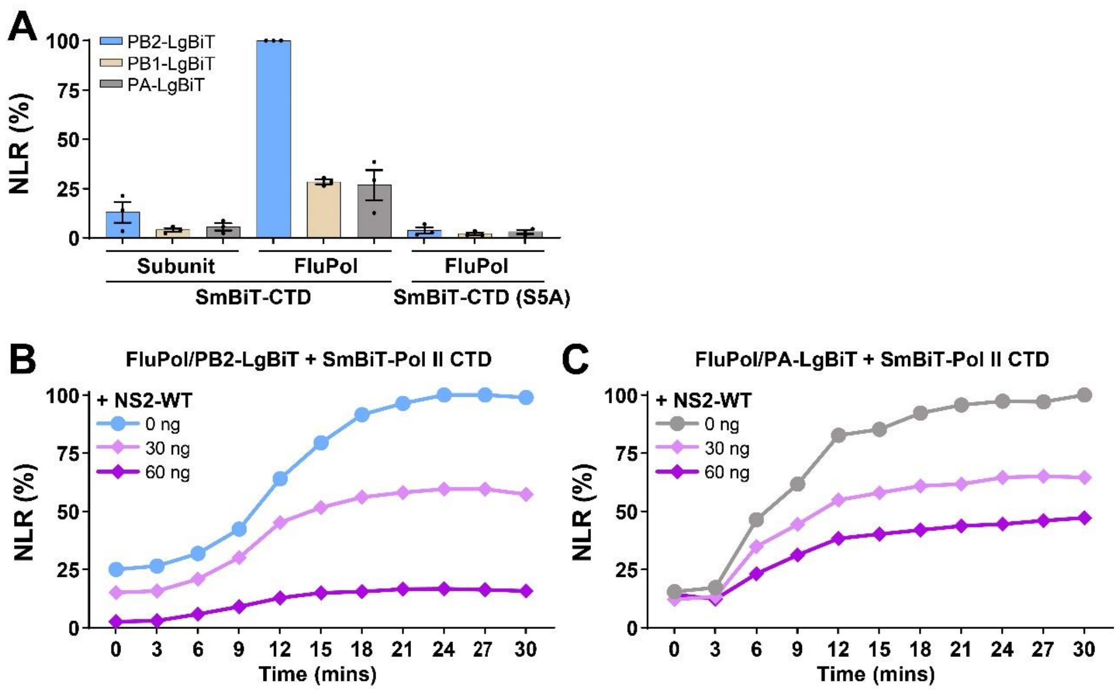

Appendix Figure S5. NS2 interferes with the interaction between FluPol and Pol II CTD in cells.

(A) Interactions between Pol II CTD and individual FluPol subunit or FluPol complex assessed by NanoBiT luciferase complementation assay. HEK-293T cells were co-transfected with the pCAGGS plasmids expressing SmBiT-tagged Pol II CTD and one LgBiT-tagged FluPol subunit alone or in combination with the other two non-tagged subunits. After 24 hpt, the Nano luciferase enzymatic activities were measured. The SmBiT-Pol II CTD (S5A), in which all serine 5 residues were replaced with an alanine, was used as negative control. (B-C) Inhibitory effects of wild-type NS2 on FluPol-Pol II CTD interaction. HEK-293T cells were co-transfected with the pCAGGS plasmids expressing SmBiT-tagged Pol II CTD and FluPol/PB2-LgBiT (C) or FluPol/PA-LgBiT (D). The 30 or 60 ng of pCAGGS plasmids expressing NS2-WT were co-transfected. After 24 hpt, the Nano luciferase enzymatic activities were measured. The signal intensities of the Nano luciferase at successive time points are shown.

Appendix Table S1. Cryo-EM data processing and refinement statistics.

	FluPol-NS2 complex (hexamer)	FluPol-NS2 complex (local refinement)
**Data collection and processing**		
Microscope	Titan Krios	Titan Krios
Detector	K3 subunit	K3 subunit
Magnification	29,000	29,000
Voltage (kV)	300	300
Electron exposure ($e^{-}/Å^2$)	60	60
Defocus range (μm)	-1.0 to -2.0	-1.0 to -2.0
Pixel size (Å)	0.69	0.69
Symmetry imposed	C1	C1
Final particle images (no.)	351,243	351,243
Map resolution (Å)	3.0-3.1	3.0
FSC threshold	0.143	0.143
**Refinement**		
Initial model used (PDB code)	6QNW, 1PD3	6QNW, 1PD3
Model resolution range (Å)	Up to 3.0	Up to 3.0
Map sharpening *B* factor ($Å^2$)	DeepEMhancer	DeepEMhancer
Map correlation coefficient		
CC_Volume	0.80	0.80
CC_mask	0.80	0.79
Model composition		
Non-hydrogen atoms	86,104	25,006
Protein residues	10,711	3,117
Nucleotide residues	-	-
B factors ($Å^2$)		
Protein	83.4	57.1
Nucleic acid	-	-
Ligand	-	-
R.m.s. deviations		
Bond lengths (Å)	0.009	0.003
Bond angles (°)	0.95	0.63
**Validation**		
MolProbity score	1.75	1.72
Clashscore	7.12	5.66
Poor rotamers (%)	0.02	0.18

Ramachandran plot		
Favored (%)	94.79	93.84
Allowed (%)	5.09	6.04
Disallowed (%)	0.11	0.13
