# Supplementary figures and images for: NS2 induces an influenza A RNA polymerase hexamer and acts as a transcription to replication switch
Source: EMBO Rep. 2024 Jul 18;25(11):8. doi: 10.1038/s44319-024-00208-4 (PMC11549089; doi:10.1038/s44319-024-00208-4)

**SourceData Fig. 2E**

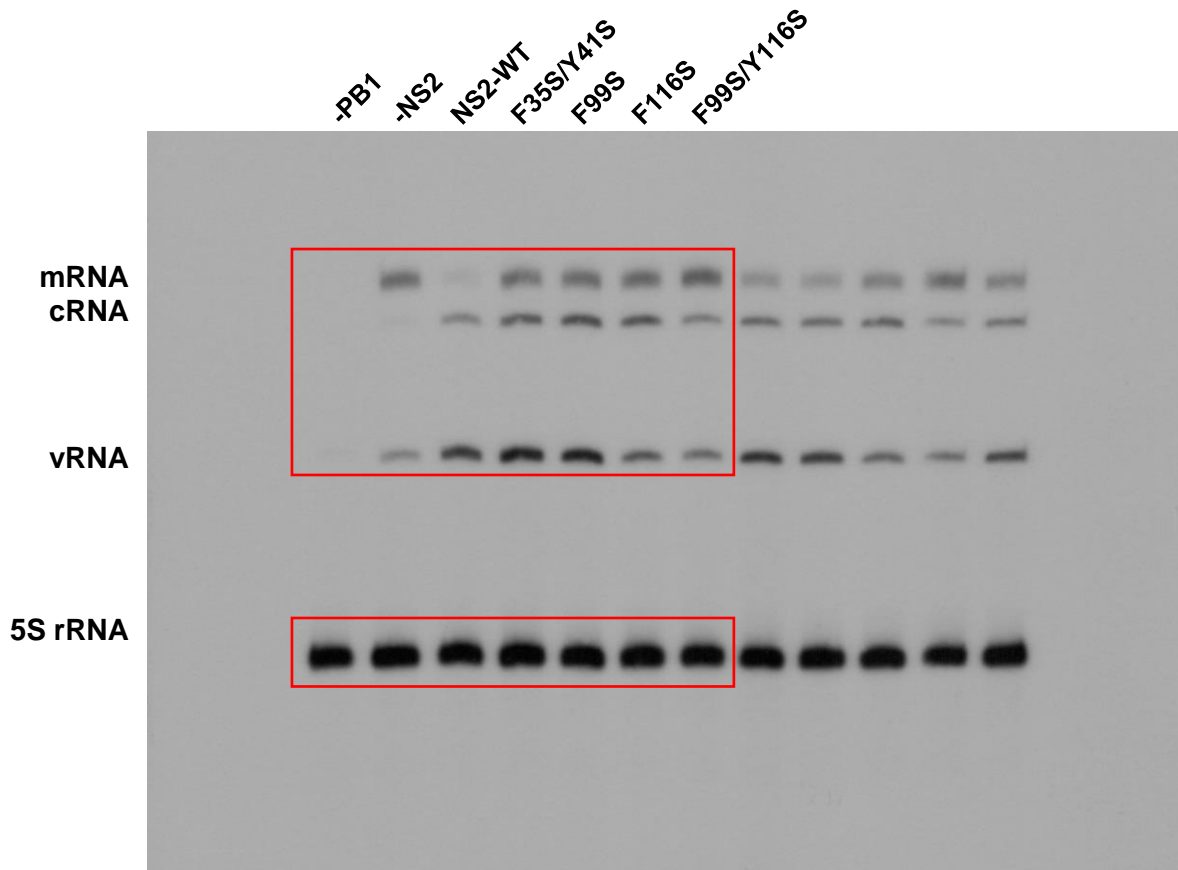

Supplement: Supplementary file 3 — Source data Fig. 2 [file 44319_2024_208_MOESM3_ESM.zip › Figure_2E_Source_Data/SourceData_2E_Effects of different NS2 substitutions on RNP activity in the mini replicon system.pdf]

SourceData Fig. 2D

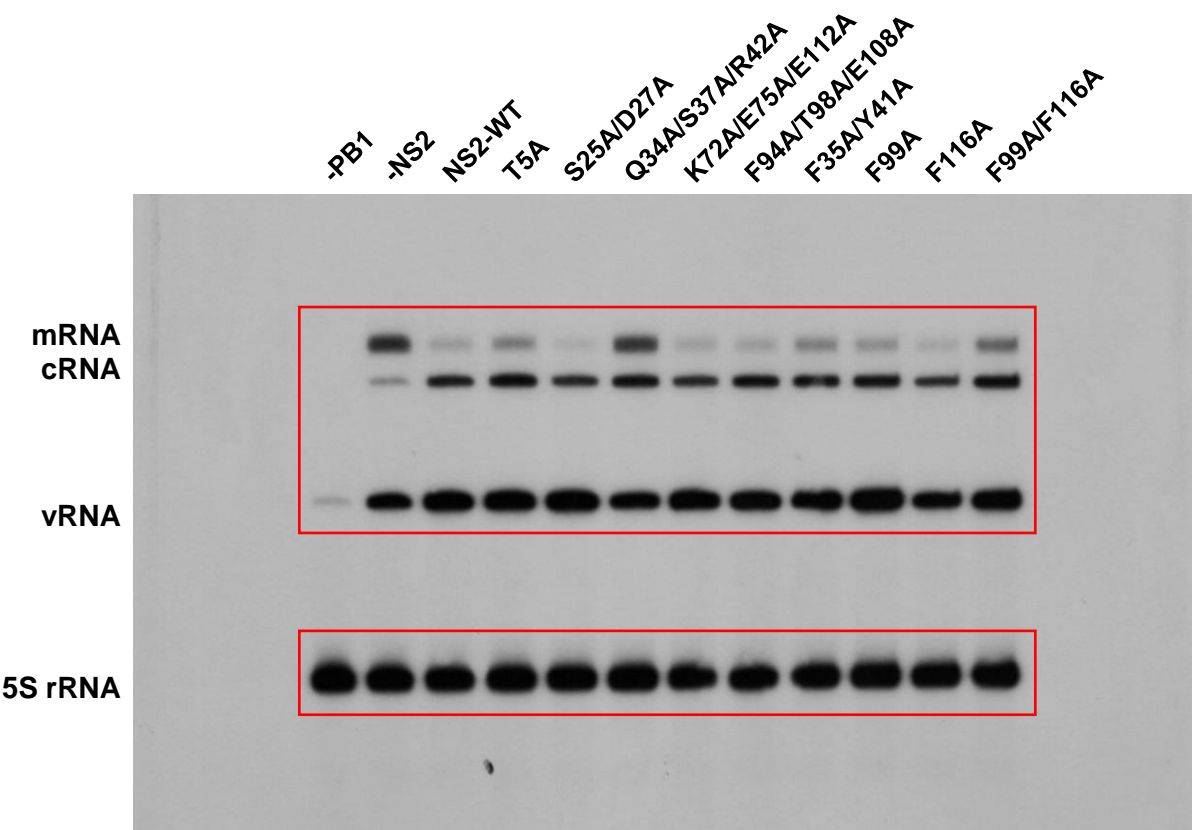

Supplement: Supplementary file 3 — Source data Fig. 2 [file 44319_2024_208_MOESM3_ESM.zip › Figure_2D_Source_Data/SourceData_2D_Effects of different NS2 substitutions on RNP activity in the mini replicon system.pdf]

SourceData Fig. 3D

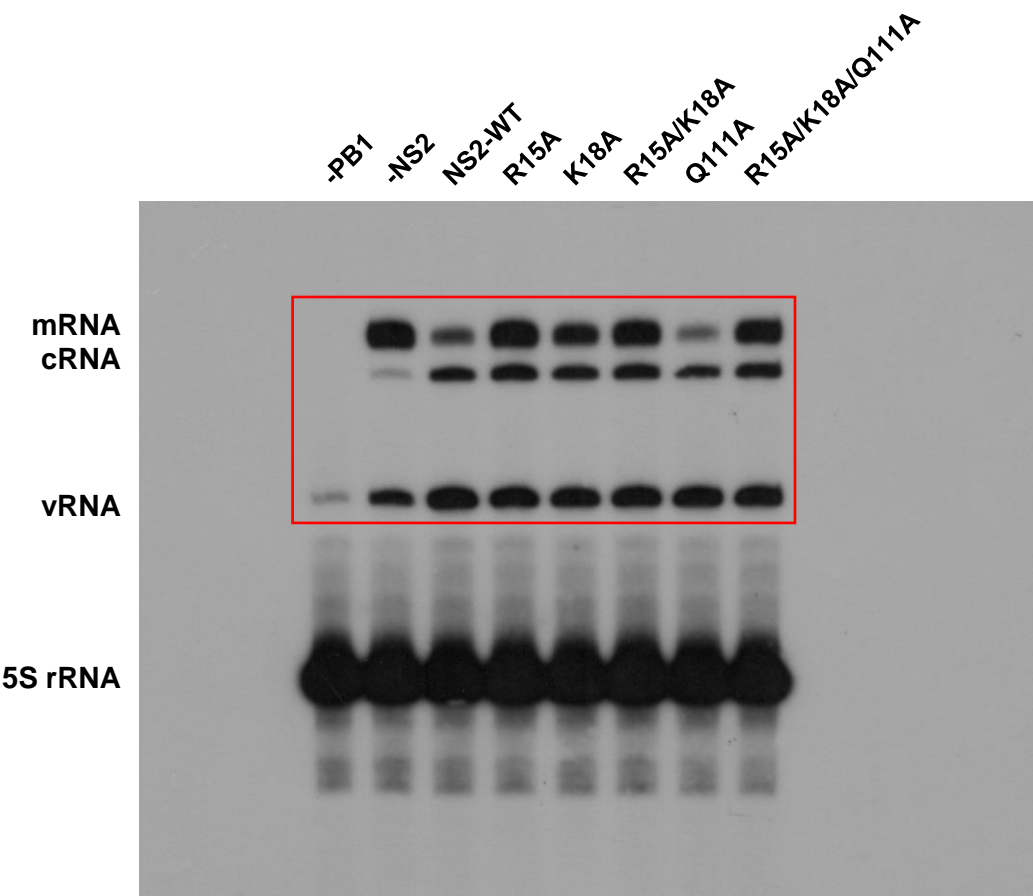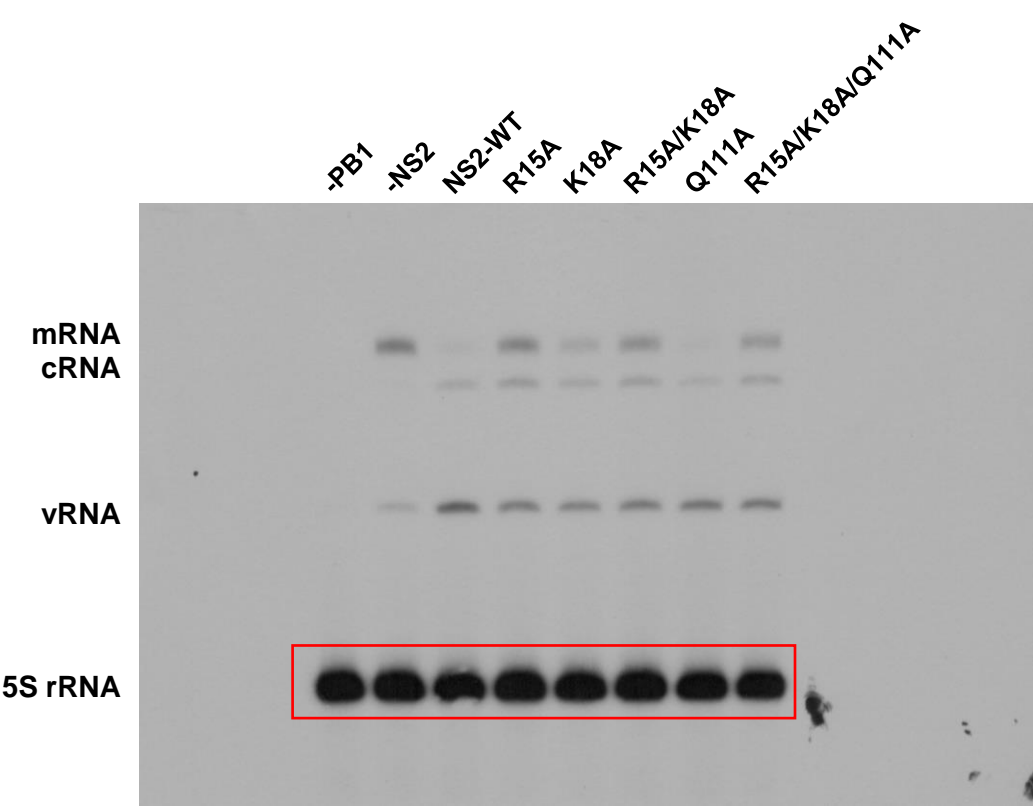

Supplement: Supplementary file 4 — Source data Fig. 3 [file 44319_2024_208_MOESM4_ESM.zip › Figure_3D_Source_Data/SourceData_3D_Effects of different NS2 substitutions on RNP activity in the mini replicon system.pdf]

SourceData Fig. 5B

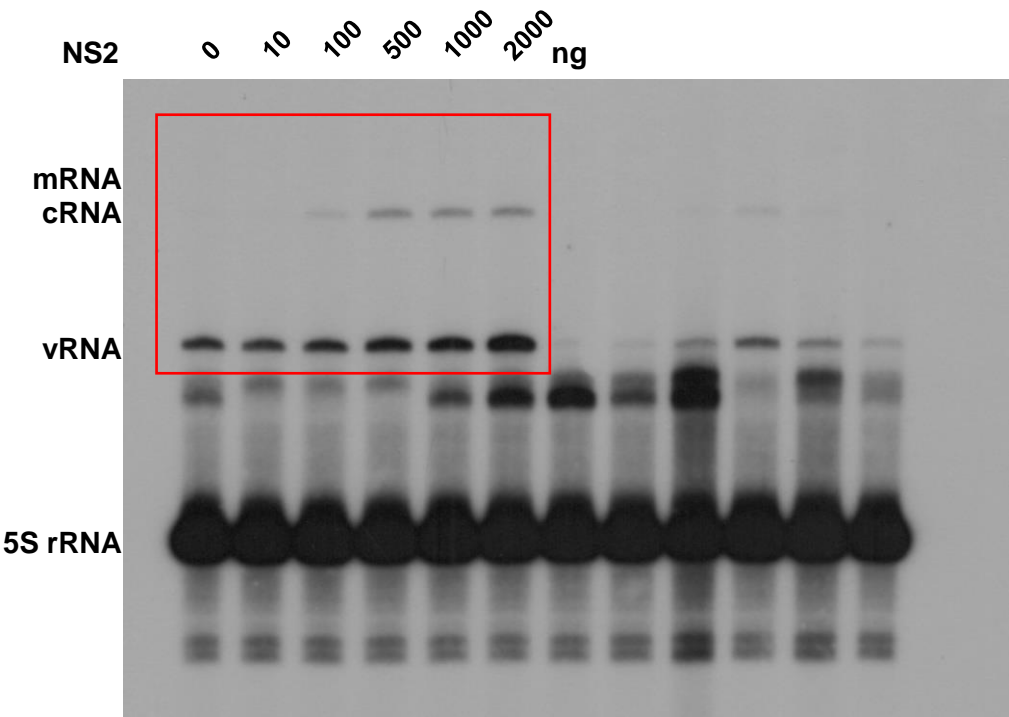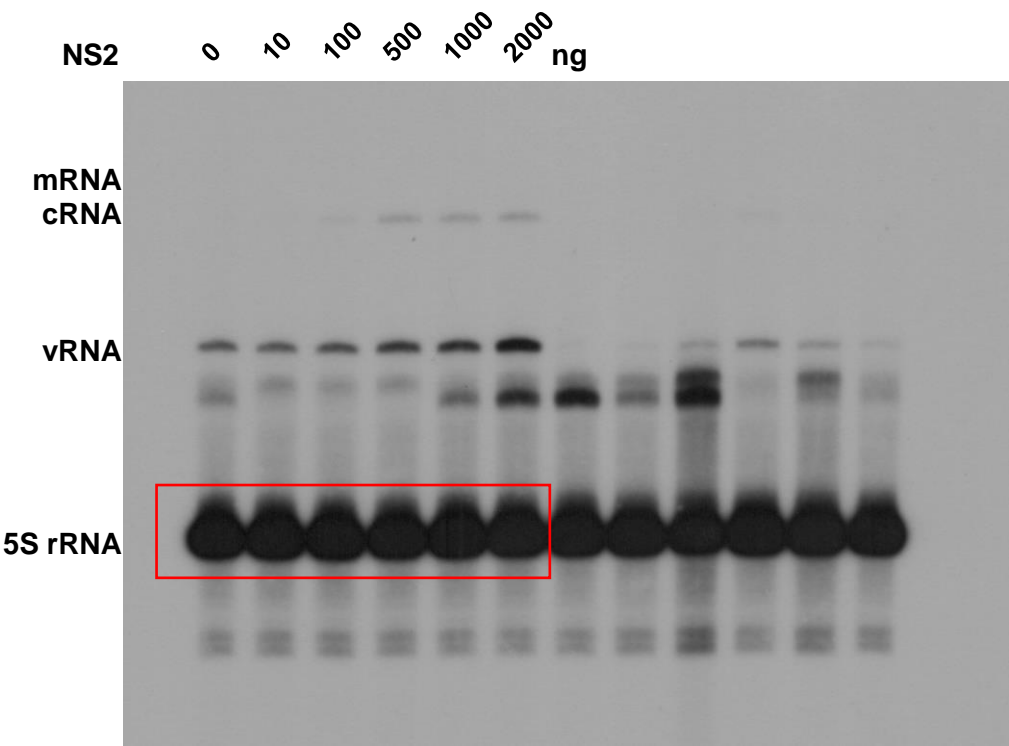

Supplement: Supplementary file 6 — Source data Fig. 5 [file 44319_2024_208_MOESM6_ESM.zip › Figure_5B_Source_Data/Figure_5B_Dose-dependent effect of NS2 on the accumulation of viral RNAs in the transcription-defective (PA-D108A) RNP reconstitution system in HEK 293T cells.pdf]
