# Supplementary material for: NS2 induces an influenza A RNA polymerase hexamer and acts as a transcription to replication switch
Source: EMBO Rep. 2024 Jul 18;25(11):8. doi: 10.1038/s44319-024-00208-4 (PMC11549089; doi:10.1038/s44319-024-00208-4)
Supplement: Supplementary file 7 — Expanded View Figures [file 44319_2024_208_MOESM7_ESM.pdf]

Expanded View Figures

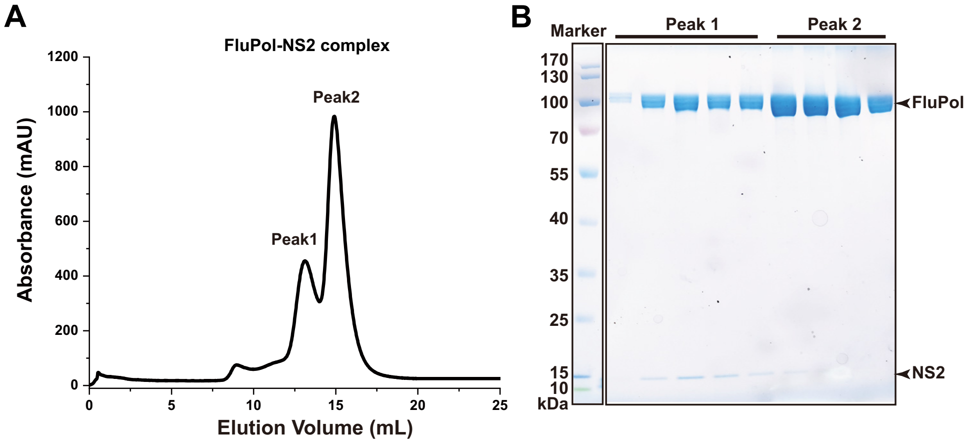

Figure EV1. Gel-filtration profile of the FluAPol-NS2 complex.

(**A**) Size-exclusion chromatography of the FluAPol-NS2 complex. (**B**) SDS-PAGE profile of the FluAPol-NS2 complex.

© The Author(s)

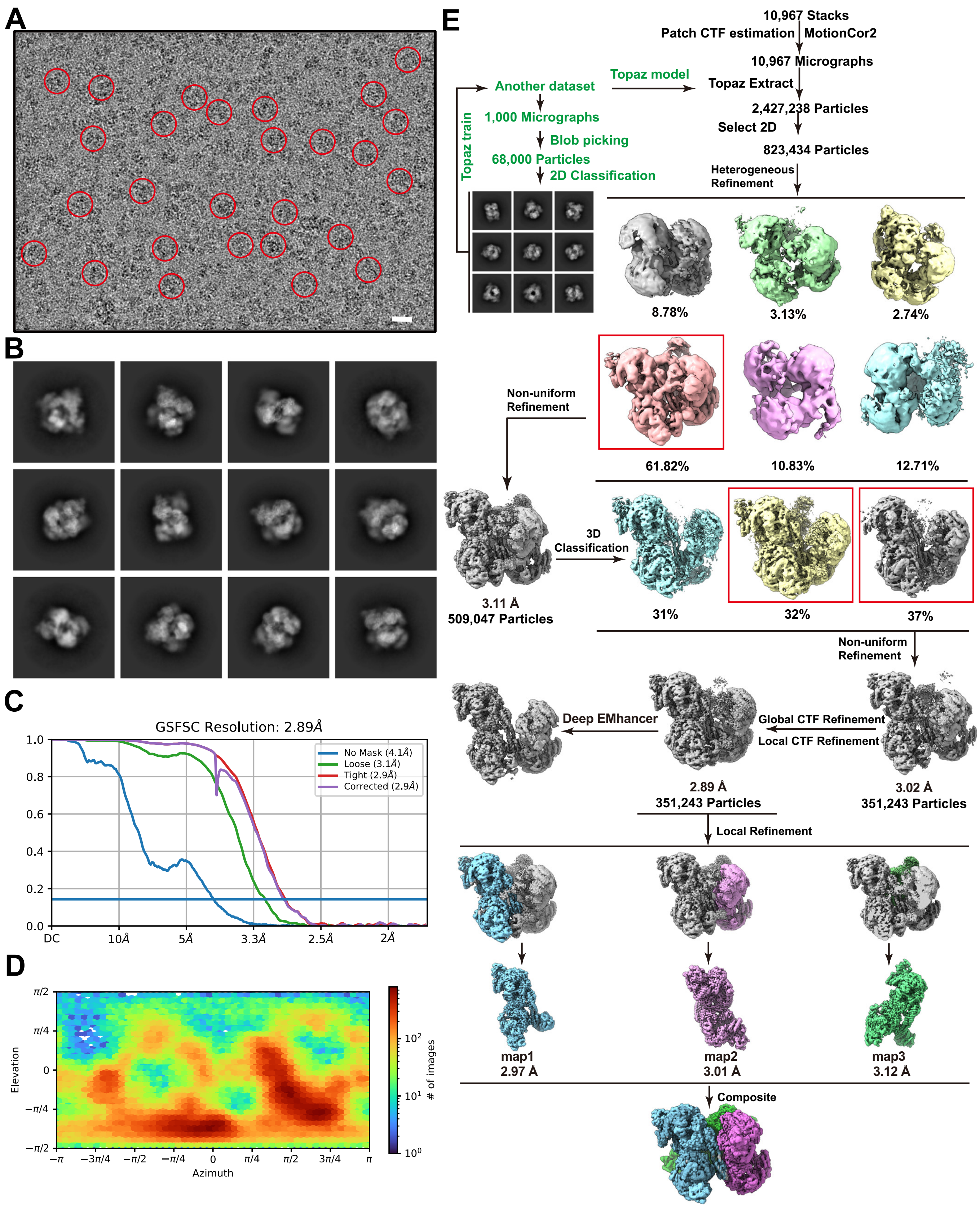
A
B
C
GSFSC Resolution: 2.89Å
No Mask (4.1Å)
Loose (3.1Å)
Tight (2.9Å)
Corrected (2.9Å)
DC
10Å
5Å
3.3Å
2.5Å
2Å
D
Elevation
Azimuth
# of images
E
10,967 Stacks
Patch CTF estimation
MotionCor2
10,967 Micrographs
Topaz model
Topaz Extract
Another dataset
1,000 Micrographs
Blob picking
68,000 Particles
2D Classification
Topaz train
2,427,238 Particles
Select 2D
823,434 Particles
Heterogeneous Refinement
8.78%
3.13%
2.74%
Non-uniform Refinement
61.82%
10.83%
12.71%
3D Classification
3.11 Å
509,047 Particles
31%
32%
37%
Non-uniform Refinement
Deep EMhancer
Global CTF Refinement
Local CTF Refinement
2.89 Å
351,243 Particles
3.02 Å
351,243 Particles
Local Refinement
map1
2.97 Å
map2
3.01 Å
map3
3.12 Å
Composite

 © The Author(s)

Figure EV2. Cryo-EM analysis of the FluAPol-NS2 complex.

(**A**) A representative cryo-EM micrograph of the FluAPol-NS2 complex is shown. The complex particles are indicated by red circles. Scale bar: 20 nm. (**B–E**) Typical 2D class average images (**B**), FSC curves (**C**), Euler angle distribution (**D**), image processing workflow (**E**) of the FluAPol-NS2 complex.

© The Author(s)

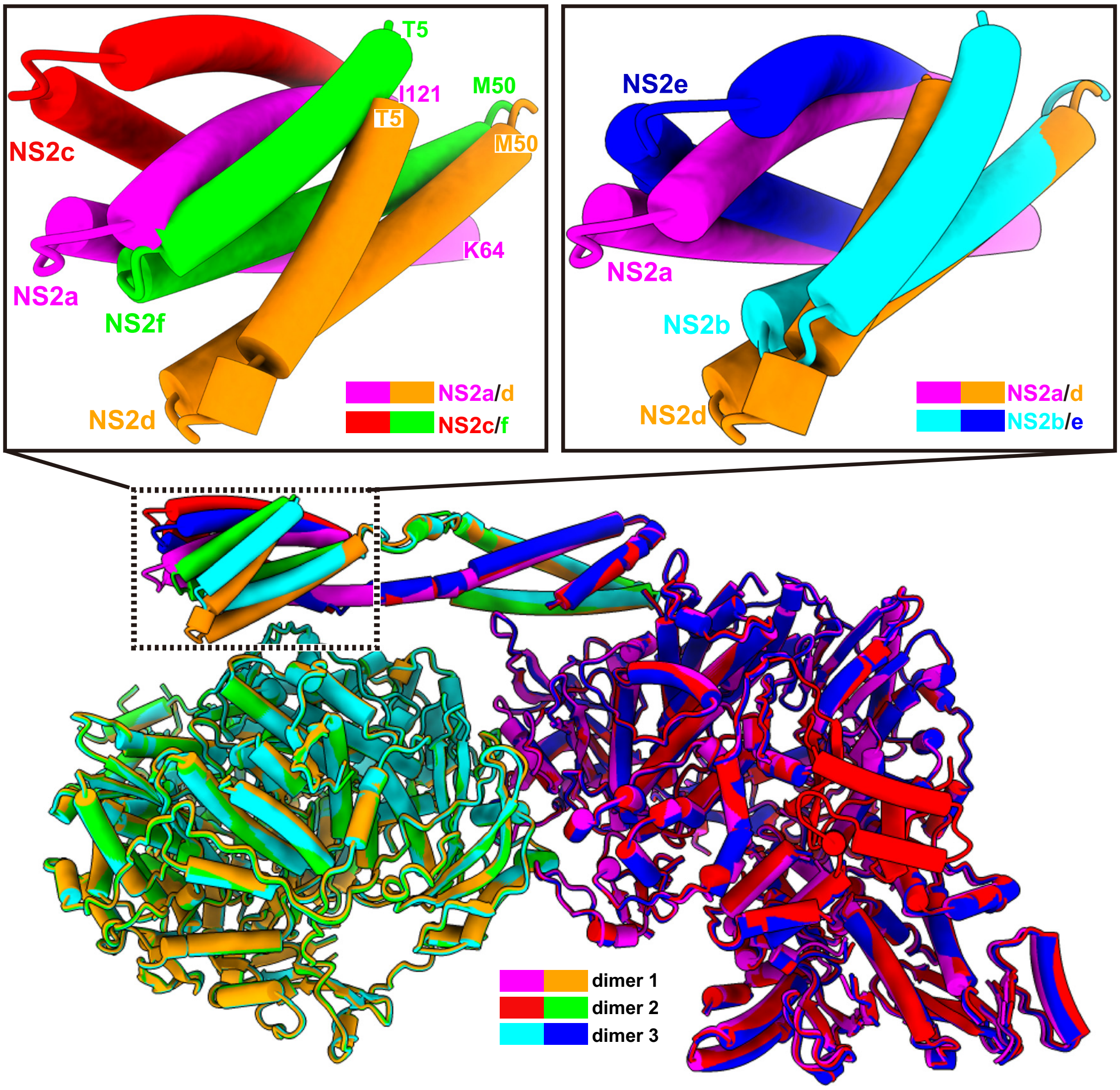

Figure EV3. Flexibility of the domain-swapped NS2 dimers.

The FluAPol-NS2 hexamer complex could be divided into three parts based on the EM density maps, each of them containing a FluAPol dimer and a domain-swapped NS2 dimer. We overlaid three parts, and three FluAPol dimers could be superimposed well. NS2 shows almost the same conformation at one end but significantly different conformation at the other end.

 © The Author(s)

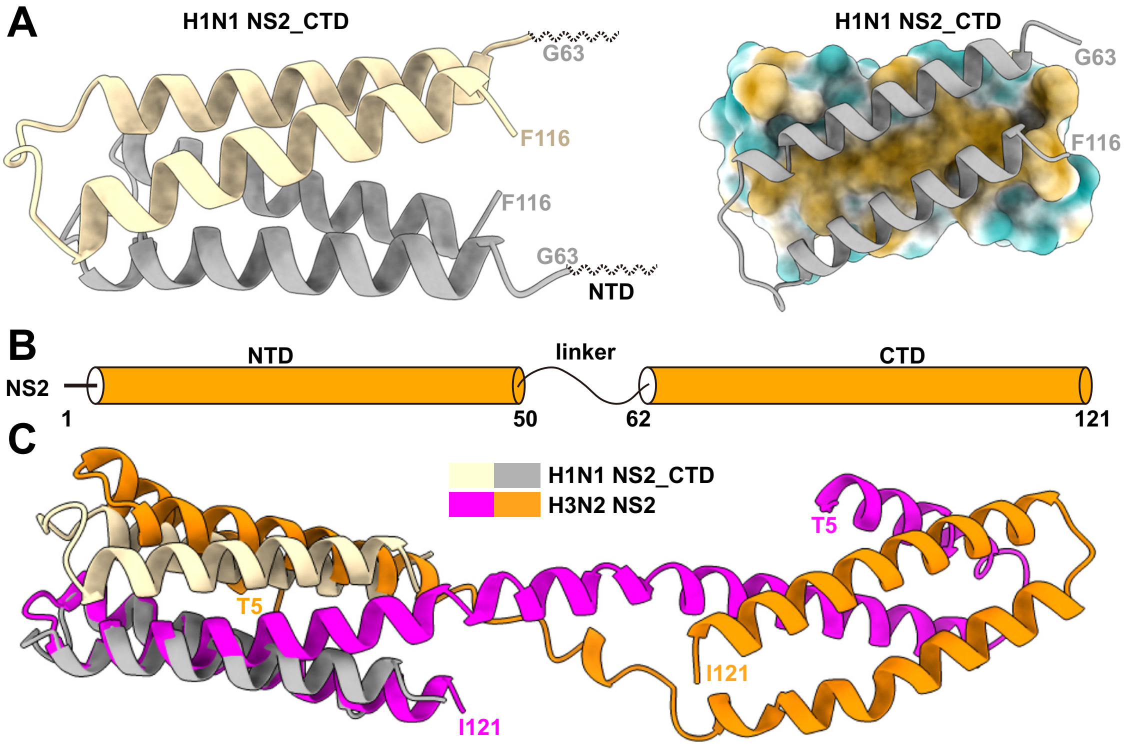

Figure EV4. Structure of the full-length influenza NS2.

(**A**) The crystal structure of the H1N1 NS2 CTD (PDB ID:1PD3) is shown. The NS2 CTD could form a dimer mainly through hydrophobic interactions. (**B**) Schematic diagram of the domains of the NS2 protein. (**C**) Structural overlay of the previously solved structure of the NS2 CTD dimer with the domain-swapped NS2 dimer solved.

© The Author(s)

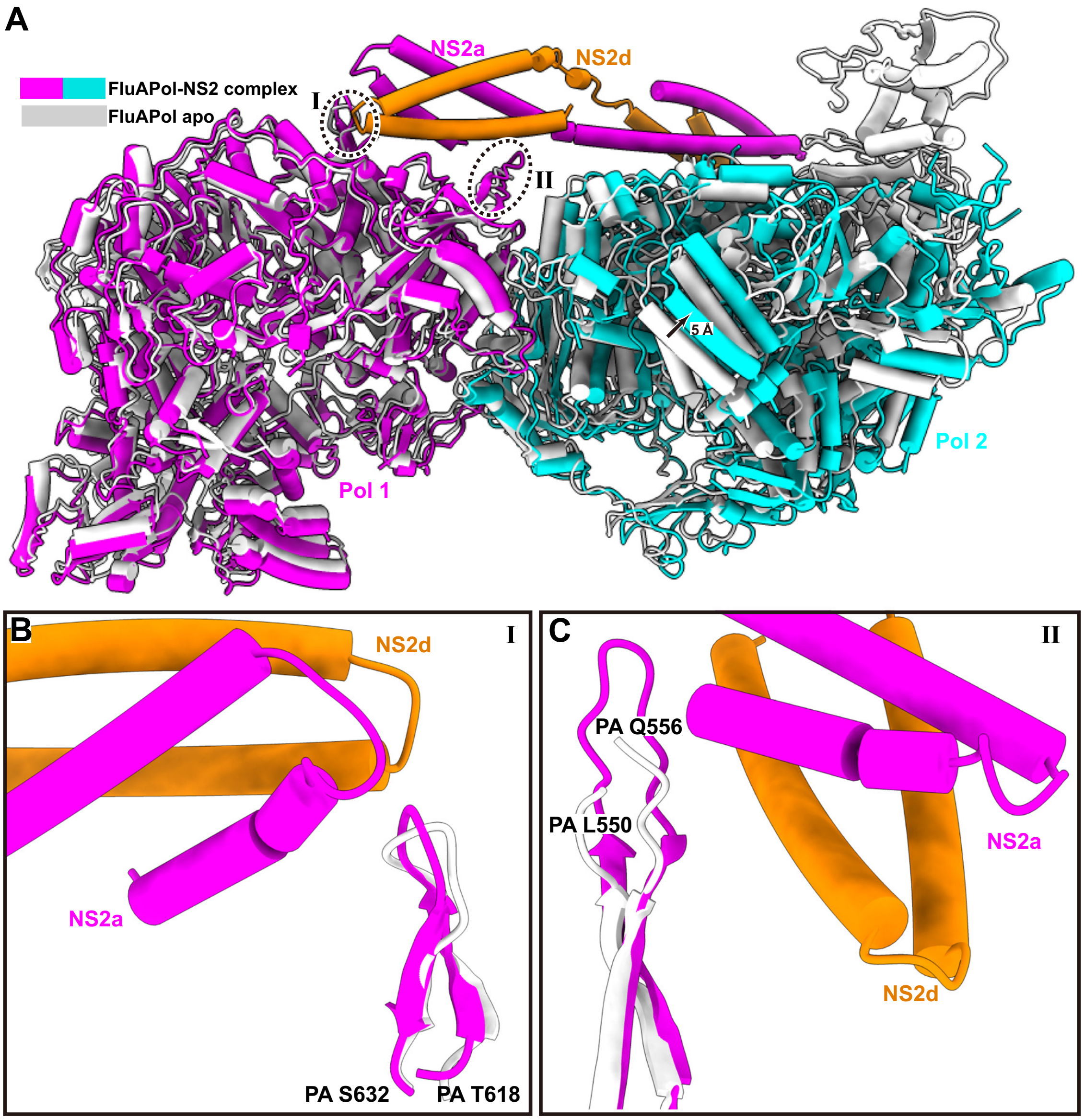

Figure EV5. Conformational changes of the influenza polymerase dimer induced by NS2 binding.

(**A**) A superimposition of structures of FluAPol-NS2 complex and apo FluAPol is shown. (**B**, **C**) Close up of site I (**B**) and II (**C**).

 © The Author(s)
